# Supplementary material for: LRP10, PGK1 and RPLP0: Best Reference Genes in Periprostatic Adipose Tissue under Obesity and Prostate Cancer Conditions
Source: Int J Mol Sci. 2023 Oct 13;24(20):15140. doi: 10.3390/ijms242015140 (PMC10606769; doi:10.3390/ijms242015140)
Supplement: Supplementary file 1 [file ijms-24-15140-s001.zip › Figure S1.pdf]

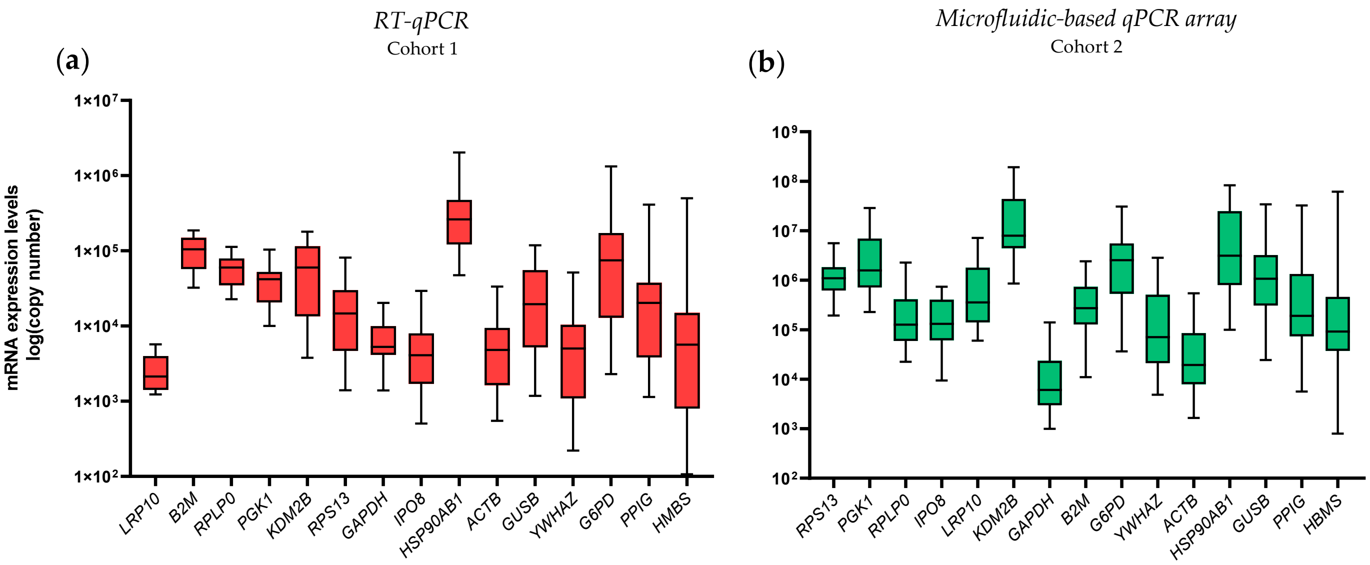

Figure S1. Absolute mRNA copy number levels of 15 evaluated internal reference genes in human PPATs. Data (Mean ± SD of copy number) obtained by RT-qPCR technique (a), or microfluidic-based qPCR array technique (b). Standard curves for each selected gen were run in parallel to quantify the absolute mRNA copy number across the samples used.
