# Supplementary material for: LRP10, PGK1 and RPLP0: Best Reference Genes in Periprostatic Adipose Tissue under Obesity and Prostate Cancer Conditions
Source: Int J Mol Sci. 2023 Oct 13;24(20):15140. doi: 10.3390/ijms242015140 (PMC10606769; doi:10.3390/ijms242015140)
Supplement: Supplementary file 1 [file ijms-24-15140-s001.zip › Figure S2.pdf]

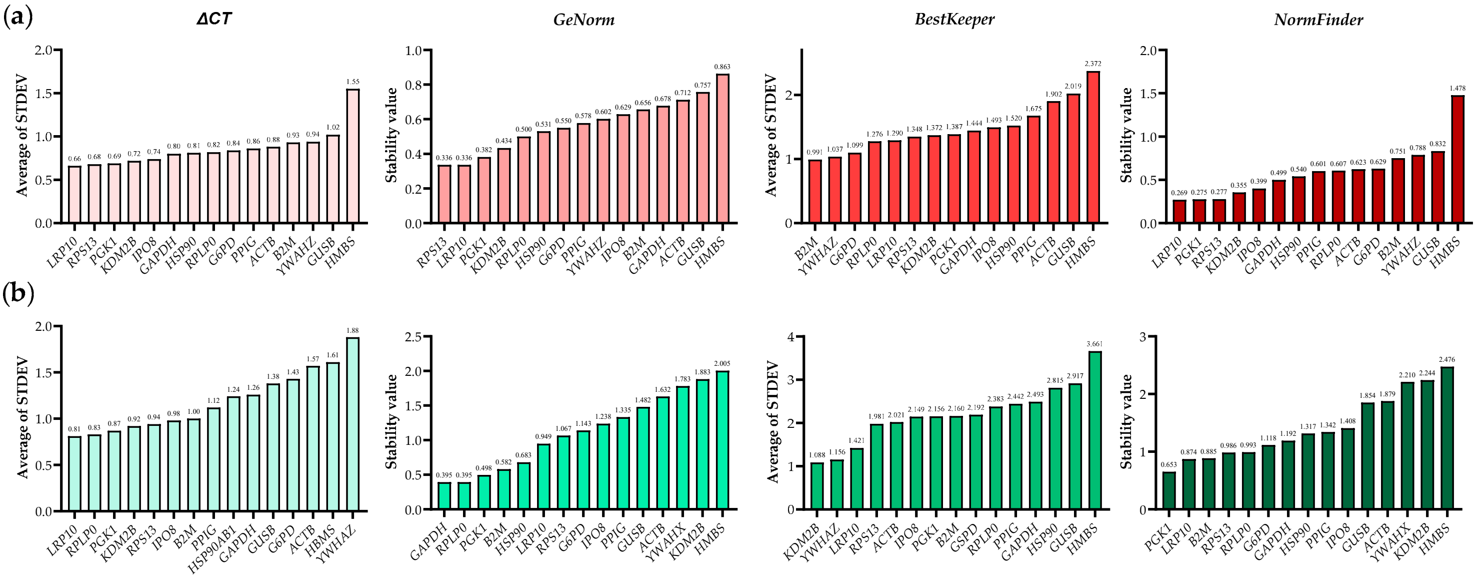

Figure S2. ΔCT method, GeNorm, BestKeeper, and NormFinder specific suitability parameters. Specific software parameters were calculated for 15 internal reference genes evaluated by: (a) RT-qPCR; and, (b) microfluidic-based qPCR array methodology.
