# Supplementary material for: LRP10, PGK1 and RPLP0: Best Reference Genes in Periprostatic Adipose Tissue under Obesity and Prostate Cancer Conditions
Source: Int J Mol Sci. 2023 Oct 13;24(20):15140. doi: 10.3390/ijms242015140 (PMC10606769; doi:10.3390/ijms242015140)
Supplement: Supplementary file 1 [file ijms-24-15140-s001.zip › Table S1.pdf]

Table S1. Statistical parameters for reference gene election across the evaluated battery of genes. SD (Standard Deviation; GN – GeNorm; BK – BestKeeper; NF – NormFinder).

Gene	Method	Min	Max	Mean	SD	GN [a]	BK [b]	NF [c]
ACTB	RT-qPCR	17.01	26.11	21.54	2.15	0.712	1.902	0.623
	Mic-Array	14.84	26.11	19.71	2.80	1.632	1.421	1.879
B2M	RT-qPCR	21.51	26.67	23.11	1.50	0.656	0.991	0.751
	Mic-Array	17.21	26.28	20.97	2.29	0.582	2.16	0.885
G6PD	RT-qPCR	19.77	27.32	23.89	2.32	0.550	1.099	0.629
	Mic-Array	15.25	24.91	19.29	2.43	1.143	2.192	1.118
GAPDH	RT-qPCR	21.88	28.79	24.45	1.87	0.678	1.444	0.499
	Mic-Array	18.71	26.81	23.33	2.26	0.395	2.493	1.192
GUSB	RT-qPCR	26.44	33.61	29.54	2.16	0.757	2.019	0.832
	Mic-Array	17.63	28.89	22.92	3.01	1.482	2.917	1.854
HMBS	RT-qPCR	21.64	34.57	29.02	3.17	0.863	2.372	1.478
	Mic-Array	14.27	31.49	23.73	3.46	2.005	3.661	2.476
HSP90AB1	RT-qPCR	18.86	26.52	23.65	2.06	0.531	1.52	0.540
	Mic-Array	15.38	25.40	20.02	2.91	0.683	2.815	1.317
IPO8	RT-qPCR	27.16	33.41	30.43	1.92	0.629	1.493	0.399
	Mic-Array	22.19	28.90	25.01	2.02	1.238	2.149	1.408
KDM2B	RT-qPCR	29.62	35.3	31.66	1.78	0.434	1.372	0.355
	Mic-Array	19.36	27.32	23.40	2.20	1.883	1.088	2.244
LRP10	RT-qPCR	25.73	30.72	27.99	1.47	0.336	1.290	0.269
	Mic-Array	16.01	22.98	19.84	2.17	0.949	2.021	0.874
PGK1	RT-qPCR	23.78	29.73	26.64	1.76	0.382	1.387	0.275
	Mic-Array	16.36	23.47	20.19	1.99	0.498	2.156	0.653
PPIG	RT-qPCR	24.76	34.04	30.17	2.61	0.578	1.675	0.601
	Mic-Array	17.88	28.57	24.78	3.03	1.335	2.442	1.342
RPLP0	RT-qPCR	23.36	28.97	25.97	1.71	0.501	1.276	0.607
	Mic-Array	14.36	21.41	18.42	2.01	0.395	2.383	0.993
RPS13	RT-qPCR	22.65	28.9	25.58	1.81	0.336	1.348	0.277
	Mic-Array	16.13	23.74	19.21	1.91	1.067	1.981	0.986
YWHAZ	RT-qPCR	24.16	30.93	26.73	2.21	0.602	1.037	0.788
	Mic-Array	18.13	26.01	21.60	2.48	1.783	1.156	2.21

[a] Expression stability value M

[b] Cq standard deviation

[c] Expression stability value
