# Supplementary material for: LRP10, PGK1 and RPLP0: Best Reference Genes in Periprostatic Adipose Tissue under Obesity and Prostate Cancer Conditions
Source: Int J Mol Sci. 2023 Oct 13;24(20):15140. doi: 10.3390/ijms242015140 (PMC10606769; doi:10.3390/ijms242015140)
Supplement: Supplementary file 1 [file ijms-24-15140-s001.zip › Table S2.pdf]

Table S2. Gene symbol, primers sequences, Amplicon Size (A.S.), official name, and function of validation measured genes.

GENE	SEQUENCE	A.S (BP)	OFFICIAL NAME	FUNCTION
LEP	GGTGACTTTCTGTTTGGAGGAG<br>GTGGCTTTGGCCCTATCTTT	142	Leptin	Adipocyte-secreted cytokine that plays a major role in the regulation of energy homeostasis
ANGPT1	GAAGGGAACCGAGCCTATTC<br>TTTTCCTGCTGTCCCAGTGT	102	Angiopoietin 1	Member of a glycoprotein family involved in vascular development and angiogenesis
MMP2	CTACGATGGAGGCGCTAATG<br>ACTCTTTGTCCGTTTTGGGG	150	Matrix Metallopeptidase 2	Gelatinase A, type IV collagenase enzyme capable of cleaving extracellular matrix components
MMP9	CAGTGCCATGTAAATCCCCA<br>CACCTCCACTCCTCCCTTTC	102	Matrix Metallopeptidase 9	Extracellular matrix remodeling enzyme that degrades type IV and V collagens
