# Supplementary material for: LRP10, PGK1 and RPLP0: Best Reference Genes in Periprostatic Adipose Tissue under Obesity and Prostate Cancer Conditions
Source: Int J Mol Sci. 2023 Oct 13;24(20):15140. doi: 10.3390/ijms242015140 (PMC10606769; doi:10.3390/ijms242015140)
Supplement: Supplementary file 1 [file ijms-24-15140-s001.zip › Table S3.pdf]

**Table S3.** Bibliographic search for the selection of internal reference genes in periprostatic adipose tissues**.** Unless otherwise indicated, studies are referred to human tissues.

| Ref. | Tissue | Evaluated genes |
|---|---|---|
| [1] | Adipocytes | *ACTB, B2M, GAPDH, HMBS* |
| [2] | Adipocytes | *GAPDH, GUSB* |
| [3] | AT - not specified | *ACTB, B2M, GAPDH, GUSB, LRP10, RPLP0, YWHAZ* |
| [4] | AT - not specified (mouse) | *G6PD* |
| [5] | AT - not specified (mouse) | *ACTB, GAPDH, HSP90AB1* |
| [6] | AT - not specified (mouse) | *ACTB, B2M* |
| [7] | BAT | *B2M, GAPDH, IPO8, GUSB, HMBS, LRP10, PGK1, RPLP0, YWHAZ* |
| [8] | BAT, PGAT (mouse) | *ACTB, B2M, PPIG* |
| [9] | BAT, PGAT, SAT (mouse) | *GAPDH* |
| [10] | BAT, WAT | *ACTB, GAPDH* |
| [11] | EPAT | *ACTB, B2M, GAPDH, RPLP0, PPIG* |
| [12] | EPAT, PRAT, iWAT, BAT (mouse) | *ACTB, B2M, GAPDH, RPLP0, PPIG, YWHAZ* |
| [13] | iWAT (mouse) | *B2M, HMBS* |
| [14] | SAT, OMAT | *ACTB, B2M, GAPDH, HMBS, IPO8, RPLP0, PGK1, YWHAZ* |
| [15] | SAT, OMAT | *ACTB, B2M, GAPDH, GUSB, PGK1, RPLP0* |
| [[16] | SAT, PGAT (mouse) | *ACTB, B2M, GAPDH, GUSB* |
| [17] | SAT, VAT (rat) | *ACTB, B2M, GAPDH, HSP90AB1, GUSB* |
| [18] | Universal | *RPS13* |
| [19] | Universal | *ACTB, B2M, GAPDH, RPS13* |
| [20] | Universal | *ACTB, B2M, GAPDH, G6PD, GUSB, HMBS, IPO8, RPLP0, RPS13* |
| [21] | Universal | *RPLP0* |

AT – Adipose Tissue; BAT – Brown Adipose Tissue; iWAT – Inguinal White Adipose Tissue; SAT – Subcutaneous Adipose Tissue; VAT – Visceral Adipose Tissue; EPAT – Epididymal Adipose Tissue; PRAT – Perirenal Adipose Tissue; OMAT – Omental Adipose Tissue; PGAT – Perigonadal Adipose Tissue; EPAT – Epicardial Adipose Tissue; WAT – White Adipose Tissue

**REFERENCES**


1. Cahyadi, D.D., T. Warita, N. Irie, K. Mizoguchi, J. Tashiro, Y.Z. Hosaka, and K. Warita, *Housekeeping gene expression variability in differentiating and non-differentiating 3T3-L1 cells.* Adipocyte, 2023. **12**(1): p. 2235081. doi: 10.1080/21623945.2023.2235081
2. Krautgasser, C., M. Mandl, F.M. Hatzmann, P. Waldegger, M. Mattesich, and W. Zwerschke, *Reliable reference genes for expression analysis of proliferating and adipogenically differentiating human adipose stromal cells.* Cell Mol Biol Lett, 2019. **24**: p. 14. doi: 10.1186/s11658-019-0140-6
3. Gabrielsson, B.G., L.E. Olofsson, A. Sjögren, M. Jernås, A. Elander, M. Lönn, M. Rudemo, and L.M. Carlsson, *Evaluation of reference genes for studies of gene expression in human adipose tissue.* Obes Res, 2005. **13**(4): p. 649-52. doi: 10.1038/oby.2005.72
4. Anderson, J. and G. Hollifield, *Glucose-6-phosphate dehydrogenase activity in adipose tissue from two types of obese mice during starvation.* Metabolism, 1966. **15**(12): p. 1092-7. doi: 10.1016/0026-0495(66)90098-9
5. Pedersen, M.H., L. Lauritzen, and L.I. Hellgren, *Fish oil combined with SCFA synergistically prevent tissue accumulation of NEFA during weight loss in obese mice.* Br J Nutr, 2011. **106**(10): p. 1449-56. doi: 10.1017/s0007114511001917
6. Hoggard, N., M. Cruickshank, K.M. Moar, P. Barrett, S. Bashir, and J.D. Miller, *Inhibin betaB expression in murine adipose tissue and its regulation by leptin, insulin and dexamethasone.* J Mol Endocrinol, 2009. **43**(4): p. 171-7. doi: 10.1677/jme-09-0046
7. Taube, M., J.C. Andersson-Assarsson, K. Lindberg, M.J. Pereira, M. Gäbel, M.K. Svensson, J.W. Eriksson, and P.A. Svensson, *Evaluation of reference genes for gene expression studies in human brown adipose tissue.* Adipocyte, 2015. **4**(4): p. 280-5. doi: 10.1080/21623945.2015.1039884
8. Fromm-Dornieden, C., O. Lytovchenko, S. von der Heyde, N. Behnke, S. Hogl, J. Berghoff, F. Köpper, L. Opitz, U. Renne, A. Hoeflich, T. Beissbarth, B. Brenig, and B.G. Baumgartner, *Extrinsic and intrinsic regulation of DOR/TP53INP2 expression in mice: effects of dietary fat content, tissue type and sex in adipose and muscle tissues.* Nutr Metab (Lond), 2012. **9**(1): p. 86. doi: 10.1186/1743-7075-9-86

9. Wu, Y., J.Y. Kim, S. Zhou, and C.M. Smas, *Differential screening identifies transcripts with depot-dependent expression in white adipose tissues.* BMC Genomics, 2008. **9**: p. 397. doi: 10.1186/1471-2164-9-397
10. Wargent, E.T., J.F. O'Dowd, M.S. Zaibi, D. Gao, C. Bing, P. Trayhurn, M.A. Cawthorne, J.R. Arch, and C.J. Stocker, *Contrasts between the effects of zinc-α2-glycoprotein, a putative β3/2-adrenoceptor agonist and the β3/2-adrenoceptor agonist BRL35135 in C57Bl/6 (ob/ob) mice.* J Endocrinol, 2013. **216**(2): p. 157-68. doi: 10.1530/joe-12-0402
11. Chechi, K., Y. Gelinas, P. Mathieu, Y. Deshaies, and D. Richard, *Validation of reference genes for the relative quantification of gene expression in human epicardial adipose tissue.* PLoS One, 2012. **7**(4): p. e32265. doi: 10.1371/journal.pone.0032265
12. Fan, X., H. Yao, X. Liu, Q. Shi, L. Lv, P. Li, R. Wang, T. Tang, and K. Qi, *High-Fat Diet Alters the Expression of Reference Genes in Male Mice.* Front Nutr, 2020. **7**: p. 589771. doi: 10.3389/fnut.2020.589771
13. Gong, H., L. Sun, B. Chen, Y. Han, J. Pang, W. Wu, R. Qi, and T.M. Zhang, *Evaluation of candidate reference genes for RT-qPCR studies in three metabolism related tissues of mice after caloric restriction.* Sci Rep, 2016. **6**: p. 38513. doi: 10.1038/srep38513
14. Hurtado del Pozo, C., R.M. Calvo, G. Vesperinas-García, J. Gómez-Ambrosi, G. Frühbeck, R. Corripio-Sánchez, M.A. Rubio, and M.J. Obregon, *IPO8 and FBXL10: new reference genes for gene expression studies in human adipose tissue.* Obesity (Silver Spring), 2010. **18**(5): p. 897-903. doi: 10.1038/oby.2009.374
15. Catalán, V., J. Gómez-Ambrosi, F. Rotellar, C. Silva, A. Rodríguez, J. Salvador, M.J. Gil, J.A. Cienfuegos, and G. Frühbeck, *Validation of endogenous control genes in human adipose tissue: relevance to obesity and obesity-associated type 2 diabetes mellitus.* Horm Metab Res, 2007. **39**(7): p. 495-500. doi: 10.1055/s-2007-982502
16. Almeida-Oliveira, F., J.G.B. Leandro, P. Ausina, M. Sola-Penna, and D. Majerowicz, *Reference genes for quantitative PCR in the adipose tissue of mice with metabolic disease.* Biomed Pharmacother, 2017. **88**: p. 948-955. doi: 10.1016/j.biopha.2017.01.091
17. Yuan, J.C., T. Yogarajah, S.K. Lim, G.B. Yvonne Tee, and B.Y. Khoo, *Pilot study and bioinformatics analysis of differentially expressed genes in adipose tissues of rats with excess dietary intake.* Mol Med Rep, 2020. **21**(5): p. 2063-2072. doi: 10.3892/mmr.2020.11012
18. Rashid, M., S.G. Shah, A. Natu, T. Verma, S. Rauniyar, P.B. Gera, and S. Gupta, *RPS13, a potential universal reference gene for normalisation of gene expression in multiple human normal and cancer tissue samples.* Mol Biol Rep, 2021. **48**(12): p. 7967-7974. doi: 10.1007/s11033-021-06828-6
19. de Jonge, H.J., R.S. Fehrmann, E.S. de Bont, R.M. Hofstra, F. Gerbens, W.A. Kamps, E.G. de Vries, A.G. van der Zee, G.J. te Meerman, and A. ter Elst, *Evidence based selection of housekeeping genes.* PLoS One, 2007. **2**(9): p. e898. doi: 10.1371/journal.pone.0000898
20. Sharan, R.N., S.T. Vaiphei, S. Nongrum, J. Keppen, and M. Ksoo, *Consensus reference gene(s) for gene expression studies in human cancers: end of the tunnel visible?* Cell Oncol (Dordr), 2015. **38**(6): p. 419-31. doi: 10.1007/s13402-015-0244-6
21. Caracausi, M., A. Piovesan, F. Antonaros, P. Strippoli, L. Vitale, and M.C. Pelleri, *Systematic identification of human housekeeping genes possibly useful as references in gene expression studies.* Mol Med Rep, 2017. **16**(3): p. 2397-2410. doi: 10.3892/mmr.2017.6944
